# Narratives of pregnancy across 19 Countries: Analysis of a 1.5-billion-word news media database

**DOI:** 10.1371/journal.pone.0305866

**Published:** 2024-08-30

**Authors:** Kalla Maxine P. Sy, Ting Yu Joanne Chow, Jeannette R. Ickovics, Reuben Ng

**Affiliations:** 1 Department of Social and Behavioral Sciences, Yale School of Public Health, New Haven, CT, United States of America; 2 Yale-NUS College, Singapore, Singapore; 3 Lee Kuan Yew School of Public Policy, National University of Singapore, Singapore, Singapore; 4 Lloyd’s Register Foundation Institute for the Public Understanding of Risk, National University of Singapore, Singapore, Singapore; McGill University, CANADA

## Abstract

Pregnancy is a universal experience shaped by sociocultural contexts. News media presents a unique opportunity to analyze public narratives of pregnancy and how it differs across cultures. Our study aims to (1) identify the most prevalent overall themes in news media narratives of pregnancy across 19 English-speaking countries, and (2) compare pregnancy narratives across geographic regions. We used the largest English news media corpus that included over 30 million news articles from more than 7000 news websites across 19 countries, and extracted a one-year data subset (2019; 1.5 billion words). Of the primary search terms ‘pregnant’ and ‘pregnancy’, we collated 240,464 descriptors that met criteria of lexical proximity and semantic bonding. Thereafter, we used topic modelling to identify the five most prevalent pregnancy-related themes: (1) complications and risk, (2) crime, (3) celebration, (4) celebrity births, and (5) contraception. Although there were regional differences, themes of complications and risk were most common, comprising 39.6% of all pregnancy narratives in our big-data corpus. The second-most dominant theme was crime (20.8%). Narratives of contraception were more prevalent in Europe, North America, and Oceania (27.2–31.3%) compared to Africa and Asia (11.9–19.6%). Though the vast majority of pregnancies are healthy, themes of complications and risk dominated the news media discourse; unchecked, this may be an avenue for misinformation, stress, and anxiety. In addition, lower prevalence of contraception narratives in Africa and Asia may reflect a gap that requires the attention of policymakers in building culturally-adapted programs to promote family planning and encourage open discussions about sexual health. Results contribute to the academic repository of societal representations of pregnancy through a big-data lens, providing contextual information for future development, implementation and evaluation of localized pregnancy-related campaigns.

## Introduction

Pregnancy and childbirth are universal: with approximately 140 million births annually– 385,000 births daily [1]. The experience of pregnancy for each woman is inherently shaped by different resources, sociocultural beliefs and practices embedded across their communities and countries [2, 3]. Navigating pregnancy is complex, given the need to make decisions about care and childbirth and to negotiate the uncertainties of changing identities and relationships. Women considering or already experiencing pregnancy often look for information and advice—from friends, family, neighbors, healthcare professionals as well as mass and social media—to support their pregnancy-related decision-making [4, 5]. Mass media has social power in creating shared societal definitions, which in turn shape how individuals perceive, understand, and respond to issues and topics [6]; therefore, it is crucial to understand how pregnancy is discussed and conceptualized.

Mass media has a vital role in public discourse, functioning as a platform to access information, form judgments, and articulate opinions [7], yet research on pregnancy in mass media has been limited. Media simultaneously reflects community-level perceptions and realities, while shaping the knowledge and attitudes of community members [8]. According to Agenda-setting Theory, first proposed by McCombs and Shaw in 1968, mass media impacts the representation of topics in the public eye, not by directing people’s opinions but by making certain themes more salient than others [9, 10]. In this context, mass media can generate greater public awareness and concern for specific aspects of the pregnancy experience. This information and public discourse are now widely accessible to most of the world’s population [11, 12]. Online articles, as a dominant source of information today [13], present a unique opportunity for researchers to analyze a big-data source that encodes a representative snapshot of its time. These repositories of publications, produced within a naturalized setting [14], frame and phrase topics aligned with current social norms, hegemonic forces, and journalistic conventions, allowing researchers to retrospectively interrogate culturally-relevant discourses [15] and discussions [16].

Previous studies have investigated societal understandings of pregnancy by examining individual subjective experiences primarily through self-report questionnaires [17, 18], in-depth interviews [19], and focus groups [20]. Such qualitative methods have enabled researchers to gain deep insights into women’s individual narratives of pregnancy, contextualizing their experiences against a backdrop of their socio-cultural and identity subgroups. To contribute to this repository of pregnancy literature, our study adopts a complementary approach to the qualitative inquiry, by checking for representativeness [21]—testing its applicability in a wider big-data context. We interrogate pregnancy narratives beyond the individual, expanding into broader society by leveraging easy access to online content and harnessing machine learning techniques that have become increasingly relevant to analyzing the massive and continuously growing data at low cost [22]. Topic modelling, specifically, Latent Dirichlet allocation (LDA), is one technique that can be leveraged to identify major thematic clusters in textual data by establishing latent semantic patterns between words across documents [23]. LDA previously has been applied in public health to understand public conversations about mental illness [24–26], vaccinations [27], the Covid-19 pandemic [28–30], and ageism [31–37]: findings derived from interrogating media coverage and content are valuable in guiding policy recommendations for topic framing [38], topic representations [39], and further advocacy [40] against a socially-aware backdrop [41].

Within the specific domain of pregnancy, a recent study by Wexler and colleagues [42] applied machine learning models to analyse the content in online birth forums, revealing fears about maternal health complications and pregnancy risks as a dominant discussion theme among expectant mothers. The investigators concluded that women use these forums to seek emotional and peer support as well as to meet unmet informational needs across the perinatal period. This study provided important insights, though was limited to a focus on information pregnant women actively sought from peers during pregnancy on online forums. To date, no study has systematically identified the broader mass media discourse about pregnancy, nor have any studies examined potential regional differences.

The primary objective of this study is to investigate emergent narrative themes surrounding pregnancy by applying LDA to a vast dataset from 19 country-specific mass media outlets published in 2019, where English is used as a medium of publication and official language. The specific aims are to: (1) identify the most prevalent themes in the overall narratives of pregnancy and within specific geographical regions, and (2) compare the prevalence of narrative themes across regions.

Through the interrogation of published discussions and conceptualizations of pregnancy, this study elucidates emergent narratives found in the media. Regionally-differentiated results of narrative prevalence may be used as a springboard to guide follow-up localized studies. As we identify gaps in media coverage on family planning information across geographies, this could in turn provide food for thought on developing and implementing targeted public policy programs, contextualized by thematic contexts identified in our study.

## Materials and methods

### Dataset

The News on the Web (NOW) Corpus [43] is the largest international corpus of the English language, including more than 28 million online-published articles from more than 7000 news websites and platforms across 19 countries. Funded by the National Science Foundation and the National Endowment for the Humanities, the dataset was developed to study contemporary language usage in countries where English is widely used. The platform hosts a dynamic corpus that retrieves publicly-available news data by searching Google News for newly-listed newspaper and magazine articles covering a wide range of topics from current events, technology, lifestyle, and entertainment. A user may download the corpus, with textual data containing metadata (date, title, source, country of publication) for analytical purposes.

Geographical regions include North America (United States, Canada), Europe (Ireland, United Kingdom); Oceania (Australia, New Zealand); Asia (Bangladesh, Hong Kong, India, Malaysia, Pakistan, Philippines, Singapore, Sri Lanka); Africa (Ghana, Kenya, Nigeria, South Africa, Tanzania). This study extracted the full-year dataset of 2019 from the corpus, accounting for approximately 1.5 billion words included in this analysis.

### Corpus pre-processing

The 2019 corpus was prepared for analysis by identifying all articles containing any of our target terms—‘pregnant’ and ‘pregnancy’. These target keywords were selected to capture and interrogate whole-of-society narratives on the general experience of pregnancy and articles about pregnant individuals. To keep the corpus as societally-representative as possible, the investigators opted to omit keywords containing specific terminology like ‘obstetrics and gynaecology’, to avoid introducing a skew toward articles in the medical field, which may predispose results toward medicalization. Investigators also omitted semantically-adjacent terms like ‘maternity’ or ‘motherhood’ from the keyword list, to avoid introducing *post-*pregnancy narratives like neonatal care, childrearing experiences and maternity leave, which fall adjacent to the research scope.

The 2019 pregnancy corpus was pre-processed by means of lemmatisation and tagging for parts-of-speech, used to clean and filter the dataset, removing non-content words, including—but not exhaustively—common proper nouns, personal pronouns, conjunctions, determiners, cardinals, forms of ‘have’ and ‘be’ (finite/infinitive), thus only retaining content-heavy words (i.e., adjectives, nouns, verbs, adverbs) for meaningful collocate analysis. Data pre-processing analyses were conducted using Python 3.7 and OriginPro 2019b.

Next, collocates (commonly co-occurring descriptors) were identified and shortlisted based on two stringent qualifying criteria: Lexical Proximity, whereby the collocate appears within six words prior to, or after our target term. For instance, within these 3 sample texts:

1. “…told The New York Times that 16 women and children, including two **pregnant** women, were killed in the revenge attack…” (*The New York Times*, 11 July)
2. “…followed by eclampsia, a condition in which one or more convulsions occur in a **pregnant** woman suffering from hypertension, and abortion-related complications…” (*The Daily Star*, 18 Jun)
3. “…others use abortion pills to bring **pregnancy** to an end; these women are criminalised—they risk arrest and prosecution…” (*Belfast Live*, 24 Jan)

Data pre-processing will have removed non-content words (indicated in strikethrough font); and retained collocational candidates (indicated in underlined font) within a relevant proximity of ±6 words to our target keywords pregnant/pregnancy (indicated in bold font).

Next, these collocational candidates were assessed using their Mutual Information (MI) Score, a measure of semantic bonding; a score of three and above indicates that the collocate had a stronger association with our target pregnancy term. This MI score is calculated using formulae $\frac{\log\left[\frac{C*SizeCorpus}{A*B*Span}\right]}{\log\;2}$, where ‘A’ denotes the frequency of our target pregnancy keyword; ‘B’ frequency of the collocate in the corpus; ‘C’ frequency of ‘A’ and ‘B’ appearing together in joint collocation; ‘Size’ refers to the number of words in our corpus; ‘Span’ set at 12 under the criteria of 6 words to the left and right of our target keyword.

In short, the higher the MI value, the closer the relationship between target pregnancy keyword (A) and collocate (B); indicating semantic proximity and suggesting a higher chance of appearing together rather than separately. This methodologically-validated [44] and rigorous selection process culminated in 240,464 shortlisted collocates for further analyses.

### Latent dirichlet allocation (LDA) and labelling

Latent Dirichlet Allocation (LDA) is a generative probabilistic statistical model that uses natural language processing and computational linguistics to study stereotypic valence—whether a topic is predominantly positively- or negatively- connoted; encoded in mainstreamed journalistic conventions—and topic content [22, 23]. It is a rigorous approach grounded in machine learning; originating in evolutionary biology, and has been applied to numerous areas of medicine and public health increasingly in the past few years [e.g., 24–42]. Our pre-processed corpus was put through this methodology to perform topic extraction on these collocates [45]. LDA assumes that each document in a dataset contains topics defined by a distribution of certain words [46], and calculates statistical correlations among the words in a corpus dataset to generate clusters of words with the highest probability of appearing together [47].

For instance, using our three sample texts for illustrative purposes, semantically-meaningful collocational candidates of pregnancy included: (1) *killed*, *revenge*, *attack*—theme of violence toward pregnant women; (2) *eclampsia*, *condition*, *convulsions*, *suffering*, *hypertension*, *complications*, *abortion-related*—theme of pregnancy-related complications; (3) *abortion*, *pills*, *end*, *criminalised*, *risk*, *arrest*, *prosecution*—theme of access to contraception. This process is scaled up using LDA: applied to our large-scale dataset, the method assesses whether latent topical patterns probabilistically emerge across all pregnancy articles. Upon successful execution, this generated clusters of high-probability words are labelled by a human coder to best describe their composition and themes. To define topic labels, the lead author independently inspected the top 20 words that were representative of the resulting 10 topics for each geographical region and assigned labels that best described each topic cluster. Assigned topic labels were reviewed by the other investigators, achieving 97% consensus. Any labelling disagreement was resolved through discussion and a review of the ten articles most strongly associated with a given topic until arriving at a final agreement. All then collaborated to categorize each topic into overall, broad categories.

## Results

The analyses revealed a rich set of themes related to pregnancy and birth. The five most prevalent narrative themes were identified from the topic clusters: (1) complications and risk, (2) crime, (3) celebration, (4) celebrity births, and (5) contraception. The theme of complications and risk in pregnancy narratives refers to complications in pregnancy and the risks of developing complications in pregnancy. High-probability words categorized under this theme included *complication*, *risk*, *difficult*, *disease*, *miscarriage*, and *ectopic*. The theme of crime refers to felonies involving pregnant women, which included key words such as *victim*, *police*, *rape*, *kill*, *murder*, and *shoot*. The theme of celebration refers to the significance of pregnancy for a couple through acts of news sharing. High-probability words categorized in the celebration theme included *announce*, *reveal*, *share*, *post*, and *couple*. The theme of celebrity births refers to any pregnancy-related news of public figures which included key words such as *actress*, *duchess*, *prince*, and *royal*. The theme of contraception refers to the prevention of unintended pregnancy or the termination of pregnancy. High-probability words under this theme included *contraception*, *pill*, *prevent*, *unwanted*, *unplanned* and *abortion*. A list of sample sentences from each theme may be found in the supporting information S1 Table for additional context.

Overall, across our 19-country data scope, themes of complications and risk comprised 39.6% of all pregnancy-related information online. The second-most dominant theme was crime (20.8%), followed by themes of celebration (11.3%), celebrity births (10.9%), and contraception (8.9%).

There are some notable differences when examining pregnancy-related narratives across geographic regions. Narratives of complications and risk associated with pregnancy were the most prevalent across all geographic regions, except Europe. Specifically, one-third to nearly one-half of all pregnancy narratives in the published corpus were about birth complications and risks: North America (33.7%), Oceania (36.8%), Asia (46.6%), and Africa (48.3%). Within Europe, narratives of complications and risk were second (20.8%) to narratives related to contraception (31.3%).

Narratives of crime were the second most prevalent overall; however, there were differences regionally. Narratives of crime were most prevalent in Oceania (25.2%), Africa (20.3%), and Europe (17.8%). This was the least prevalent category in Asia with only 9.9% of collocates reflected here; similarly, narratives related to pregnancy and crime were less prevalent in North America (12.7%). Narratives of celebration were the second-most prevalent theme in Asia (18.6%). In other regions, however, narratives of celebration had lower occurrences, with a prevalence of 13.2% in North America and 9.4% in Europe. Narratives of celebration were not detectable as a recurring topic in Africa or Oceania.

Narratives of celebrity births were most prevalent in Europe (20.6%), and considerably less frequent in the other regions, ranging from 10.9% in Oceania to 13.0% in Asia. It is worth noting that 2019 was the year that Meghan Markle and Prince Harry, Duchess and Duke of Sussex, had their first child [48], which may explain the considerably large volume of celebrity-related content in the pregnancy narratives in Europe.

**Fig 1:**
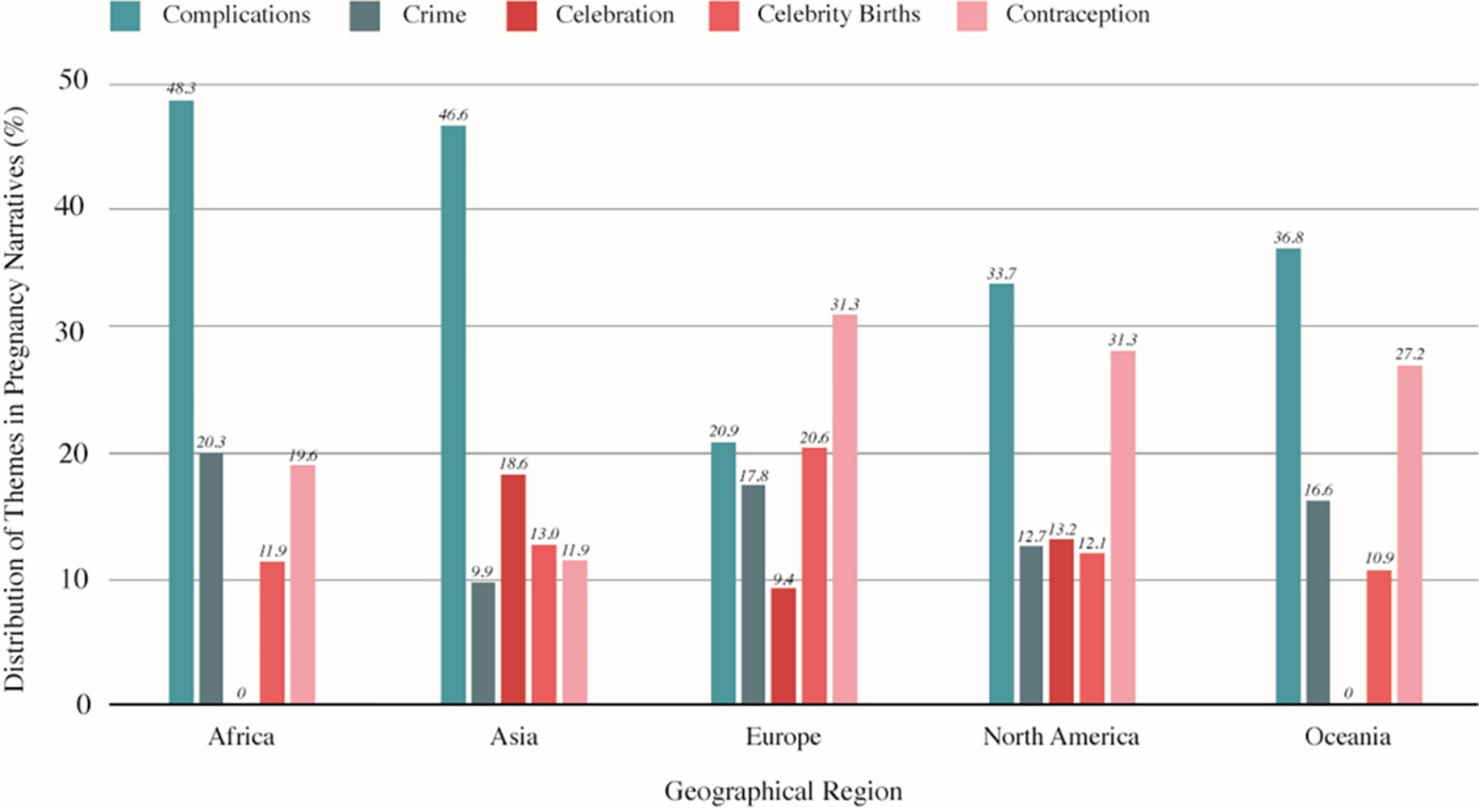
Distribution of themes in pregnancy narratives from online mass media in 2019, by geographical region.

Finally, narratives of contraception were most diverse across the five regions in terms of frequency. While contraception as a theme represented fewer than 10% of narratives in our overall big-data corpus, it represented nearly one-third (31.3%) of pregnancy narratives in Europe. The theme of contraception was also prevalent in pregnancy narratives in North America (31.3%) and Oceania (27.2%), and relatively less prevalent in Africa (19.6%) and Asia (11.9%). This is illustrated in Fig 1.

## Discussion

Pregnancy is a universal phenomenon, but how it is conceptualized and discussed in public discourse varies considerably depending on cultural context. Previous research has mainly focused on qualitative experiences; this study expands the scope by analyzing more than 28 million online-published texts across 19 countries. Through topic modelling analyses, we identified the most prevalent themes among pregnancy narratives in mass media across 19 countries and regionally.

Results demonstrate that narratives of complications and risk dominated conversations related to pregnancy. This finding contradicts and is disproportionate to the reality that most pregnancies in 2019 were healthy and uncomplicated. For example, not diminishing disproportionately high rates of morbidity and other adverse outcomes in some geographic regions, the global rate of preterm birth (an important indicator of adverse pregnancy outcomes with long-term health consequences) is 11%, with most babies born full-term (89% at >37 weeks gestation, [49]). In addition, with resources in the past decade aimed at reducing perinatal mortality, fewer than 1% of pregnancies result in maternal death [50], and fewer than 2% of births result in newborn death [51]. Nonetheless, a dominant focus on complications and risk in pregnancy narratives seems to suggest a bias toward pregnancy-specific fear and anxiety among the public [52].

This corroborates previous research that suggests that parents are most concerned about seeking information regarding pregnancy complications and risks along with guidance on how to mitigate risks to deliver a healthy child [36, 53, 54]. Parents may seek such information from online sources as often as, or more than, they do from healthcare providers [55]. The large volume of online-published mass media related to complications and risk may be indicative of an over-medicalization of pregnancy, whereby the risks associated with pregnancy and childbirth are magnified [56–58].

Although prevalence varied across regions, crime was the second-most prominent theme in pregnancy narratives overall. The relatively high prevalence of violent crime is consistent with previous research that established its overrepresentation in the news compared to its actual occurrence [59–61]. Mass media often presents a distorted version of crimes with a substantial bias towards violence to provoke public interest and to attract audience attention [62, 63].

Another striking finding is the greater prevalence of narratives of contraception in Europe, North America, and Oceania compared to those in Africa and Asia. Such trends are echoed in qualitative literature that report low media exposure to contraception narratives in these regions. For instance, varying sociocultural norms discourage open discussions on sexual health in official sources (e.g., newspapers, magazines, educational leaflets), especially in rural areas [64], with overarchingly poor access to contraceptive knowledge across sub-Saharan Africa [65] and rural Asian territories [66, 67]. Women face barriers to receiving high-quality reproductive health information and services [68], having to anonymously seek information elsewhere to avoid social shame [69]. Studies set within these regions call for greater dissemination of family planning information through mass media [70] given that media exposure was found to significantly be associated with awareness of reproductive health issues [71], likelier uptake of contraception [72], and facilitate social discussions and diffusion of knowledge by means of dispelling contraceptive myths and overcoming societal stigma [73, 74]. Our study corroborates these findings, and provide additional context for further studies aiming to build culturally-adapted programs sensitive to cross-cultural differences [75] to increase awareness and address women’s health in these regions.

There are some limitations to this study. The corpus dataset was limited to English-written media and to countries that use English as an official language. Furthermore, the study’s use of the word ‘regional’ was adopted as shorthand to describe available data from the following world areas: North America (United States, Canada), Europe (Ireland, United Kingdom); Oceania (Australia, New Zealand); Asia (Bangladesh, Hong Kong, India, Malaysia, Pakistan, Philippines, Singapore, Sri Lanka); Africa (Ghana, Kenya, Nigeria, South Africa, Tanzania). Therefore, regional conclusions are not meant to be taken as representative of the *whole* region, but rather the data from the listed countries within that region. This provides impetus for follow-up studies to source mainstream mass media data across a range of more countries, or text written in different languages.

This study was limited to a one-year time-period, specifically focused on mass media published in 2019. This year was selected as the most optimal baseline snapshot available, due to data collection limitations (insufficient data for 2023 and 2022 as of writing), and free from extenuating circumstances that may have skewed analysis (authorial impetus to avoid pandemic years 2020 and 2021; as Covid-19 was known to have briefly but significantly altered news narratives [76]). This leaves room for follow-up studies applying the same methodology to more contemporary years.

The study’s investigators also acknowledge that not all 7000+ sources of online mainstream mass media sources are equal in quality and repute, given that websites vary in the platform’s publishing ethos, adherence to journalistic conventions, and external influences like sponsorships, business or political ties. The authors rely on the amalgamation of large-scale data to circumvent individual discrepancies, using big data as representational of current social trends of describing pregnancy within the overall zeitgeist.

The study also did not include social media content. In recent years, social networking sites have been popularized as platforms for people to easily gather information and express reactions, thoughts, and opinions across a variety of topics, including pregnancy [77–79]. Given the ubiquitous use of social media globally, exclusion of these analyses provides a limited overview of societal-level pregnancy narratives.

However, this study also has notable strengths. It makes important contributions to the literature as the first to describe public narratives of pregnancy using a large-scale dataset containing multiple countries, comparing across different regions, offering a broad overview of pregnancy-related narratives. We extend the literature by analyzing pregnancy representations in online mass media from 19 countries and across published newspapers, magazines, and website articles. Methodologically, this study reveals how topic modelling can easily reveal emergent themes in pregnancy narratives on a large scale and can similarly be applied to understand other societal phenomena. With increasing democratization and access to web-based textual datasets and machine learning tools, researchers may easily interrogate societal narratives on a large scale; though results still require prudent manual labelling. Adept interpretation relies on researchers who have rigorously consulted existing literature and methodologically robust frameworks.

Future research should include social media data, which may reveal different dominant themes in pregnancy narratives and broad insights into people’s interactions with each other. Research also should examine longitudinal shifts in pregnancy-related narratives. Other directions could opt for close-reading on the textual level, to highlight granular cross-cultural journalistic differences in media framing, linguistic choices, and specific descriptors used in articles across different countries. Most interesting might be the analyses of how conceptualizations of pregnancy in mass media impact maternal well-being and decision-making in pregnancy. This would advance the theoretical understanding of mass media’s role in the pregnancy experience and have implications for and applications to public health.

## Conclusion

Providing effective care across the perinatal period will require a better understanding of how women, families, communities, and cultures understand and conceptualize pregnancy. The dominance of complications and risk in pregnancy narratives across our large-scale dataset is of public health concern as it may foster an overuse of medical interventions, which is associated with increased adverse clinical outcomes and decreased maternal autonomy [56–58]. A greater focus on topics with more negative sentiment such as complications, risk, and violent crime in societal narratives may cultivate unnecessary fear and exacerbate maternal stress, thereby leading to an increase in adverse birth outcomes [80–82]. Results revealed how narratives of contraception vary across regions, particularly in Africa and Asia, where family planning discourses are less frequently attested in mainstream mass media—validating established literature suggesting these differences may stem from sociocultural factors (e.g., cultural norms, social taboos)—underscoring a gap for policymakers to address.

These findings from mass media provide insights into societal conceptualizations of pregnancy. The study hopes to add to a growing scholarship repository investigating pregnancy from a societal representation standpoint, providing food for thought in policymaking and community-level maternal health interventions. Findings may also spur individuals to make an informed navigation of the vast amounts of online pregnancy-related information, armed with the knowledge that certain negative narratives on complications, risk, and crime are predominantly attested; promoting a more balanced perspective for women and their family’s emotional wellbeing when navigating such sources.

## Supporting information

S1 Table Sample sentences for additional context. Prevalent narrative themes related to pregnancy and birth.
(DOCX)
