# Supplementary material for: Narratives of pregnancy across 19 Countries: Analysis of a 1.5-billion-word news media database
Source: PLoS One. 2024 Aug 30;19(8):e0305866. doi: 10.1371/journal.pone.0305866 (PMC11364417; doi:10.1371/journal.pone.0305866)
Supplement: S1 Table — Prevalent narrative themes related to pregnancy and birth. (DOCX) [file pone.0305866.s001.docx]

**S1 Table. Sample Sentences for Additional Context**

Prevalent narrative themes related to pregnancy and birth.

| **Sample Sentences** | | *Source*, Date (2019) |
| --- | --- | --- |
| **Topic Cluster 1: Complications and Risk (39.6%)** | | |
| High-probability words categorized under this theme included *complication, risk, difficult, disease, miscarriage,* and *ectopic*; referring to complications in pregnancy and the risks of developing complications in pregnancy. | | |
| 1 | “…while she is *pregnant* the risk of **miscarriage**, **stillbirth** and **fetal hydrops**— a serious condition…” | *The Conversation UK*, 11 Jun |
| 2 | “…sick patients including a *pregnant* woman with **complications** —needed immediate transport to Pohnpei to seek critical medical treatment…” | *Saipan Tribune*, 7 Oct |
| 3 | “…to be increasing is the rate of **chronic hypertension** in women who are *pregnant*, which between the years…” | *Medical Daily*, 4 Dec |
| 4 | “…subject to more **unique risks** than others, and that is *pregnant* women. To learn more about the mother and her unborn baby…” | *Creamer Media South Africa,* 25 Apr |
| 5 | “…factor in determining the **risk of hypertension** during *pregnancy*. It has been found that *pregnant* women above the age of 35 are at a higher risk…” | *Times Now India,* 12 Sep |
| 6 | “…experienced **crippling stomach pains**, where doctors told her that she was *pregnant* but the foetus was **growing outside her uterus**…” | *Now To Love NZ,* 30 Nov |
| 7 | “…*pregnant* women at **high risk of complications** may benefit from a Mediterranean-style diet to reduce weight gain and **risk of gestational diabetes**…” | *Yahoo News UK,* 24 Jul |
| 8 | “…p*regnant* women not getting **sufficient iodine** may have children with impaired brain development & cognitive functions…” | *Times of India,* 28 Mar |
| 9 | “…in *pregnant* women, particular medical conditions which increase **the risk of complications** from the **flu**…” | *Canberra Times,* 29 Aug |
| 10 | “…an ultrasound in order to confirm that she is *pregnant* and diagnose any **complicating factors**, such as a **tubal, or ectopic**, *pregnancy*…” | *Townhall US,* 18 May |
| 11 | “…mothers-to-be. Working two or more night shifts in a week may increase a *pregnant* woman's risk of **miscarriage** the following week by around a third…” | *Asian News International,* 17 Mar |
| 12 | “…three times more likely to **die from complications** during *pregnancy* or childbirth…” | *CBS News,* 30 Dec |
| 13 | “…**Preeclampsia** is a dangerous *pregnancy* complication, one of the leading causes of **maternal** and infant **mortality and illness**…” | *Premium Times Nigeria,* 28 Apr |
| 14 | “…followed by **eclampsia**, a condition in which one or more **convulsions** occur in a *pregnant* woman suffering from **hypertension**, and abortion-related complications…” | *The Daily Star,* 18 Jun |
|  | | |
| **Topic Cluster 2: Crime (20.8%)** | | |
| High-probability words categorized under this theme included *victim, police, rape, kill, murder,* and *shoot*; referring to felonies involving pregnant women. | | |
| 1 | “…reduce **domestic violence** by widening options available to *pregnant* mothers. The fight against **gender-based violence** needs to be tackled from all fronts…” | *Daily Maverick South Africa,* 3 Dec |
| 2 | “…experienced **sexual violence.** Seven percent of women who have ever been *pregnant* have experienced **violence** during *pregnancy*…” | *The News International Pakistan,* 25 Feb |
| 3 | “…SAPD: 14-year-old boy charged in **killing** of *pregnant* teen and her mom…” | *San Antonio Express-News,* 28 May |
| 4 | “…Sheriff's office: *Pregnant* woman **fatally stabbed** in Texas …” | *MyNorthwest,* 29 Dec |
| 5 | “…many of those **killed** were women and children. *Pregnant* mothers were specially targeted…” | *LankaWeb,* 14 Sep |
| 6 | “…told The New York Times that 16 women and children, including two *pregnant* women, were **killed** in the **revenge attack**…” | *The New York Times,* 11 July |
| 7 | “…who was 18 at the time, says she had been **raped** by a gang member but that she had no idea that she was *pregnant*…” | *BBC World,* 16 Jul |
| 8 | “…victims contract **sexual diseases**, including HIV, from being raped and hundreds also become *pregnant*. National outcry over **rape**…” | *Mail & Guardian,* 11 Nov |
|  | | |
| **Topic Cluster 3: Celebration (11.3%)** | | |
| High-probability words categorized under this theme included *announce, reveal, share, post,* and *couple*; referring to the significance of pregnancy for a couple through acts of news sharing. | | |
| 1 | “…YouTuber Shay Mitchell **revealed** on social media Friday that she is six months *pregnant* with her first child and boyfriend…” | *Variety,* 17 Jun |
| 2 | “…her first *pregnancy*. She **posted a photo** of herself among other photos of other *pregnant* people. "**Congratulations** to all the expecting mothers out there,…” | *Business Insider,* 3 May |
| 3 | “…**posted an image** of a womb on Monday, triggering netizens to speculate that his wife was *pregnant* with their first child. The singer, 25, followed up with another photo…” | *The Straits Times Singapore,* 2 April |
| 4 | “…he followed this up with an adorable **selfie** of him and Charmaine's *pregnant* belly, asking whether his **followers** think the baby is a girl or a boy…” | *HotNewHipHop,* 2 Dec |
| 5 | “…told her family she was *pregnant* and later **posted on Facebook** an ultrasound and photos…” | *The London Free Press,* 16 May |
| 6 | “…In October, Kehlani would **announce** her *pregnancy*, keeping the identity of her unborn daughter's father a secret…” | *E! Online*, 23 Feb |
| 7 | “…The Love & Hip Hop: Hollywood' star - who is eight months *pregnant* - went into more details on her own **Instagram Story**…” | *Yahoo News,* 20 Nov |
|  | | |
| **Topic Cluster 4: Celebrity Births (10.9%)** | | |
| High-probability words categorized under this theme included *duchess, prince, royal, and actress*; referring to pregnancy-related news of public figures. | | |
| 1 | “…at the point of the announcement, Meghan is thought to have been around three months *pregnant*, which means the **Duke and Duchess** of Sussex could have conceived around July…” | *Daily Express,* 3 Feb |
| 2 | “…A clip of Markle when she was *pregnant* has resurfaced. In the video, the **duchess** can be seen…” | *International Business Times,* 17 Oct |
| 3 | “…The *pregnant* **Duchess** of Sussex and other members of the **royal family** have frequently been…” | *Daily Mail,* 7 Mar |
| 4 | “…level of emotional trauma, let alone when they're *pregnant*. They said Meghan is not the spoiled **Duchess** the media paints her…” | *The South African,* 8 Feb |
| 5 | “…to celebrate the 150th anniversary of the London Underground. Kate, who was *pregnant* with **Prince** George at the time, was given a badge…” | *Nine Australia,* 22 Sep |
| 6 | “…George's first day of school, instead staying home with acute morning sickness while *pregnant* with **Prince** Louis…” | *Telegraph UK,* 16 Sep |
| 7 | “…fans were in a frenzy after eagle-eared Instagrammers caught the whispered words "I'm *pregnant*" in the background of a video taken at **Khloe Kardashian**'s birthday…” | *Newshub NZ,* 30 Jun |
|  | | |
| **Topic Cluster 5: Contraception (8.9%)** | | |
| High-probability words categorized under this theme included *contraception, pill, prevent, unwanted, unplanned* and *abortion;* referring to the prevention of unintended pregnancy, or the termination of pregnancy. | | |
| 1 | “…How likely are you to get *pregnant* while on the **pill**? How effective your **contraception** is depends on the type…” | *Daily Mail,* 19 Feb |
| 2 | “…not taking the **pill** too long, because it may delay you getting *pregnant* once you're off of it, but, luckily, that's a myth…” | *Pulse Nigeria,* 21 Jun |
| 3 | “…women to make their own **decisions** about **whether and when** they wish to become *pregnant* as well as how many children they wish to have. Enabling…” | *Guardian,* 30 Jun |
| 4 | “…interest both as an abortion doula, someone who supports *pregnant* people before, during and after the decision and process to **terminate** a *pregnancy*, …” | *Chestnut Hill Local,* 17 May |
| 5 | “…The state has an interest in ensuring that every *pregnant* person in California who wants to have an **abortion** can obtain access to that care…” | *WTVR,* 15 Sep |
| 6 | “…too few providers; where to carry out **termination** of *pregnancy* (TOP); and the involvement of other staff…” | *Irish Examiner,* 11 Jan |
| 7 | “…to cobble together hundreds of dollars to **terminate** an **unwanted** *pregnancy* because public insurance won't pay. This policy deprives residents of…” | *Washington City Paper,* 17 Dec |
| 8 | “…many others use **abortion pills** to bring *pregnancy* to an end; these women are criminalised - they risk arrest and prosecution…” | *Belfast Live,* 24 Jan |
| 9 | “…**right to abortion** and to not be forced to experience *pregnancy* and birth. It is fundamentally about bodily autonomy…” | *Halifax Examiner,* 24 May |
